# Idler Compounds: A Simple Protocol for Openly Sharing Fridge Contents for Cross-Screening

**DOI:** 10.1021/acs.jmedchem.5c02354

**Published:** 2026-01-28

**Authors:** Rebecka Isaksson, Eve M. Carter, Charlotte K. Hind, J. Mark Sutton, Hazel Rudgyard, Adam H. Roberts, Christopher W. Moon, Yinuo Wang, Todd Group Researchers, Sandra Codony, Antón L. Martínez, Joanna Bacon, Matthew H. Todd

**Affiliations:** † School of Pharmacy and Structural Genomics Consortium, 4919 University College London, 29-39 Brunswick Square, London WC1N 1AX, U.K.; ‡ Antimicrobial Discovery, Development and Diagnostics, Countermeasures, Development, Evaluation and Preparedness, UK Health Security Agency, Porton Down, Salisbury SP4 0JG, U.K.; § Discovery Group, Countermeasures, Development, Evaluation and Preparedness, UK Health Security Agency, Porton Down, Salisbury SP4 0JG, U.K.; ∥ Institute of Biomedicine of the University of Barcelona (IBUB), 16724 Universitat de Barcelona, Barcelona 08028, Spain; ⊥ Laboratori de Química Farmacèutica, Facultat de Farmàcia i Ciències de l’Alimentació, Universitat de Barcelona, Av. Joan XXIII, 27-31, Barcelona 08028, Spain; # Innopharma Drug Screening Platform/Biofarma Research Group, CIMUS Research Center, Pharmacology Department, School of Pharmacy, University of Santiago de Compostela (USC) and Instituto de Investigación Sanitaria de Santiago de Compostela (IDIS), Santiago de Compostela 15782, Spain

## Abstract

Academic drug discovery laboratories tend to accumulate collections of compounds with great potential value that merely reside in fridges and freezers. Cross screening these libraries against alternative targets holds significant potential for uncovering novel hits, but in the academic setting compound collections are rarely used, and shared, in this way. We present a short guide for collecting small molecules not being actively pursued in group projects (which we term “idlers”) to establish an open compound library. We describe how a diverse subset of this library was screened against a panel of pathogens, with the resulting data made publicly available. We hope to encourage other academic groups to develop and share their own libraries of idlers, thereby maximizing the utility of existing resources, enabling new insights, and catalyzing novel research directions through open science.

## Graphical abstract

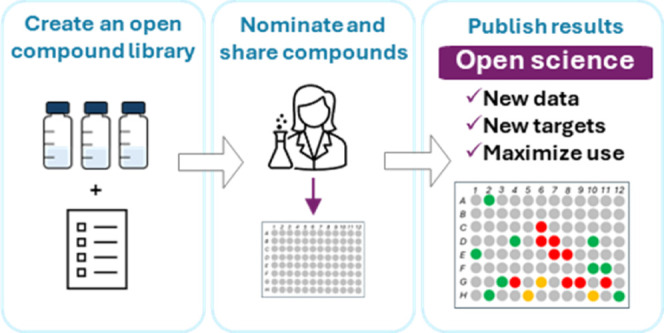

## 1 Introduction

Identifying a hit is usually the first step in a new drug discovery project. Laboratories engaged in this activity will buy, synthesize, and accumulate many prospective compounds during hit discovery and hit-to-lead projects. There is merit in cross-screening compound collections against other targets to accelerate serendipitous discovery,^1^ yet few laboratories make their compound collections available to others, either in silico or in reality. Barriers to such sharing may be legal (a desire to protect intellectual property) or logistical (the perceived difficulty of sharing physical samples with others). We propose here a simple homegrown mechanism to share compounds, termed “idlers”, commonly languishing in academic laboratory fridges and freezers, and suggest an effective way to share the associated data openly to benefit the broader community.

Compound sharing on a larger scale has been shown to be highly effective in catalyzing new discoveries toward potential therapeutics, such as the Medicines for Malaria Venture boxes^2,3^ and the Structural Genomics Consortium libraries (i.e., the Donated Chemical Probes program,^4,5^ the Kinase Chemogenomic Set^6^); in some cases, accompanying data are provided related to selectivity, toxicity, or guidelines for correct use. These compounds are made available to the community directly while stocks last, or are made available via commercial vendors, with few intellectual property restrictions, typically with the quid pro quo that the data resulting from their use should be shared, though this is challenging to enforce. These compound collections can provide a valuable alternative to industrial high throughput screening campaigns that can be beyond the reach of academic laboratories, outside of a limited number of formal collaboration arrangements that provide access to large libraries (e.g., the AstraZeneca Open Innovation platform^7^ and the now finished European Lead Factory (ELF) project^8^).

We describe the assembly of a library containing a diverse set of small molecule idler compounds that possess suitable drug-like properties derived from in-house medicinal chemistry programmes. A subset of the library was physically shared through a collaboration with the UK Health Security Agency (UKHSA); we describe the acquisition of bioactivity data for these compounds, and a method to share the data openly so that the compounds and their data may be discovered by others. By presenting our work as a “how-to”, we hope to incentivise others to share their idler compounds and openly provide the associated data.

We aim to share compounds widely; however, supplies are finite and may eventually run out. We hope that many of these compounds will be pursued further in new projects. If additional material is required, commercial compounds will likely still be available, while synthesized compounds should be easily accessible using reproducible protocols (in our case provided via the associated openly available project summaries online), which can be listed alongside compound information in the results sheet that eventuates (in the present case, in the Supporting Information). Clearly additional material would be important for determination of additional details on hits, such as IC_50_. The availability of analogues would of course be dependent on the research (and idler library assembly) carried out by the originating laboratory. For our wealthier colleagues, there are commercial solutions available for compound storage and bespoke dispensing; other solutions such as Compounds Australia^9^ may also be a suitable solution to long-term storage of samples off-site if that is desired, though we have not done this for the present work.

### 1.1 Library and Plate Design

We define idler compounds as those molecules in local storage that a team member is happy to share openly along with the associated data. To generate and share idler libraries, a few key steps are essential (Figure 1): the formation of a general compound library containing purchased and synthesized compounds from in-house medicinal chemistry programmes; the selection of a subset of diverse idler compounds from the library; providing this subset to collaborators for cross-screening; and deploying a means to share the data openly so that they are discoverable. The following sections present an overview of these steps, with further details outlined in the Supporting Information. At each step there are multiple options available; we provide here an example workflow that we have found to be effective.

**Figure 1:**
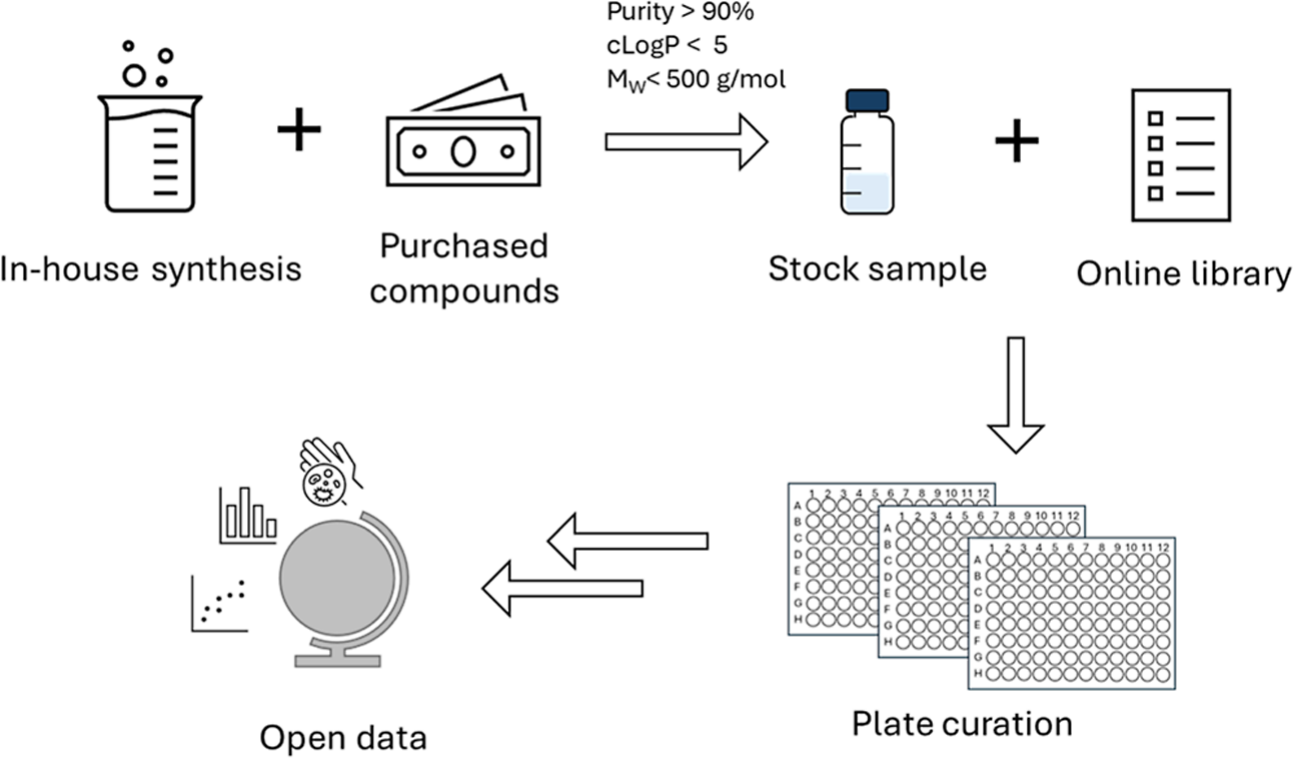
Overview of library setup and the sharing process. Compounds were synthesized or purchased for a range of projects in the research group. Idler compounds were then prepared as stock solutions and added to an online library. A subset was selected, plated and shared. A detailed step-by-step guide is provided in the Supporting Information.

### 1.2 Building the Library and Online Resources

Our library consists of small molecules relevant to ongoing drug discovery research projects, derived from both the synthetic work of the research group and commercial sources. Multimilligram quantities of pure compounds are usually made or bought, usually more than is required for evaluating the compounds for their originally intended purpose and thus leaving material that can be submitted for additional evaluation. Submitting samples of all suitable compounds to the library as a default has therefore been gradually embedded in group culture as a productive use of resources.

The physical part of the library consists of DMSO stock solutions showing >90% purity, typically judged by routine liquid chromatography mass spectrometry (LCMS); a full protocol is provided in the Supporting Information. The stock solutions are stored in vials to allow easy access to single compounds if needed for additional testing and to allow tailoring of plates when sharing compounds externally. A concentration of 10 mM was used, as this seemed a consensus choice from our biological collaborators, allowing for simple dilution in assay medium.

Electronically, the library is delineated in an online spreadsheet^10^ that contains machine-readable compound strings, along with details such as researcher/supplier origin, original target protein/project, and internal information on storage location. In parallel with the spreadsheet is an electronic laboratory notebook (ELN) containing spectroscopic data files.^11^ It is important that the spreadsheet and the ELN are trivially and openly shareable in such a way that search engines can find the data and that viewing of the data by potential users requires no authentication. We have found that Google Docs is suitable for the spreadsheet, and LabArchives^12^ possesses the desired functionality for the ELN. Any other solutions that allow such open sharing would also be suitable, and it is possible that in the future electronic repositories hosted by universities would be suitable options, given their long-term support and availability to the academic laboratories likely to generate molecules and associated data. Together, these solutions ensure good adherence to the FAIR guidelines^13^ of findability, accessibility, and interoperability (since the data may be downloaded in generic formats); these are important and widely adopted principles to help ensure the greatest impact can be derived from a data set. The reusability factor in the FAIR guidelines is ensured by a clear statement of the license associated with the data, which we would recommend to be the creative commence license CC-BY;^14^ this license allows a high level of freedom in data use with a typical academic expectation that the source of data is cited, and the license has been used across many open publishing initiatives. Making data FAIR is an excellent way to promote greater impact of the research, though there is reportedly a low level of compliance among well-known organic chemistry journals.^15^

### 1.3 Sharing Compounds from the Library

Having assembled 256 compounds in the library at the time of selection (as of June 2025, the library contains over 600 compounds), a subset of compounds was selected to fill a 96-well plate (colloquially called plate MHT-0001) as an initial collection to ship for testing. While different testing laboratories will have different preferences for plate size, a 96-well plate is a standard format widely used and for which plate translation protocols are available, enabling compounds to be moved into other formats with minimal effort; with the selection of fewer compounds, spaces could optionally be left free for controls. The selection for, and preparation of, this subset of the library took place in two steps (Figure 2). First, each researcher in the group was asked to nominate approximately five diverse compounds for inclusion; 56 compounds were selected through this process, and this provided a good opportunity for people in the group to nominate personal favorites or compounds with known biological activity that they would like to see tested more broadly. To gain as much diversity on the plate as possible, the remaining 40 slots were filled from the 156 commercially sourced compounds found in the library at the time. The commercial compounds were divided into 40 clusters (see Supporting Information) and a representative from each cluster was selected for the plate.

**Figure 2:**
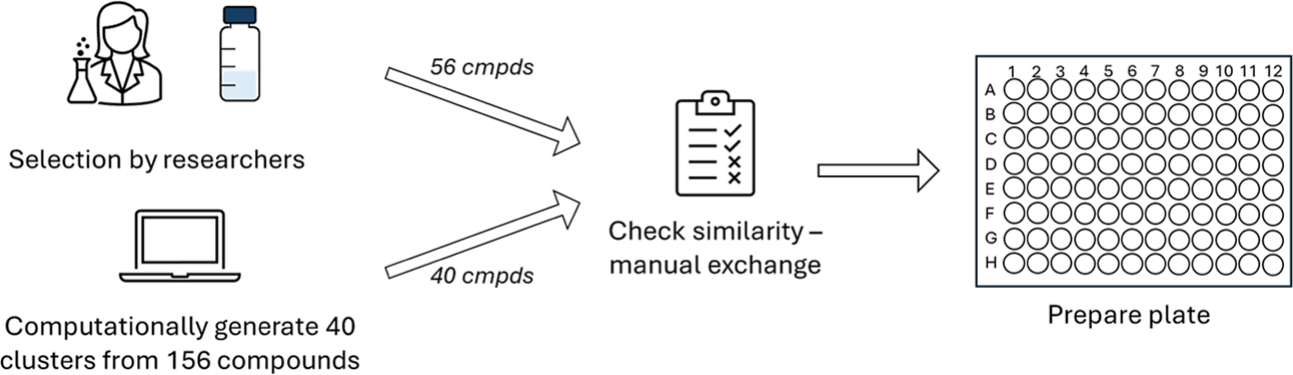
Summary of the selection process for the 96-well plate to ensure compound diversity; researchers selected 56 compounds with the remaining 40 compounds selected computationally to ensure diversity as outlined in the Supporting Information.

To ensure the full plate was diverse, a similarity check was conducted on the 96 selected compounds. This indicated six compound pairs with more than 80% similarity within each pair; after manual inspection, it was decided to exchange two of these compounds for others with greater structural variation. The 96 compounds were plated and subsequently shipped for testing at UKHSA for evaluation against a panel of pathogens.

### 1.4 Composition of the Compounds in the First Plate

The compounds shared via plate MHT-0001 were originally part of several diverse projects (Figure 3, additional material in Supporting Information). A recent focus of the Todd group has been the discovery of direct acting antivirals; 60% of the compounds were originally synthesized or purchased for such projects. Another 25% of the molecules were generated for antiparasitic subprojects as part of wider projects such as Open Source Malaria.^16^ Bacterial and human target proteins have been the focus of only a few recent projects: only 9% and 6%, respectively, of the compounds were relevant to these. The compounds selected typically had generically developable medicinal chemistry characteristics as they were part of early stage hit finding projects or were used as tool compounds for which cross screening might identify utility in another organism, such as *Plasmodium falciparum* tRNA synthetase inhibitors, where evaluation against other pathogens would be instructive. In some cases, compounds were included expressly to maximize impact of grant funding, for example compounds designed for *Onchocerca volvulus* were included to provide additional data to the relevant researcher.

**Figure 3:**
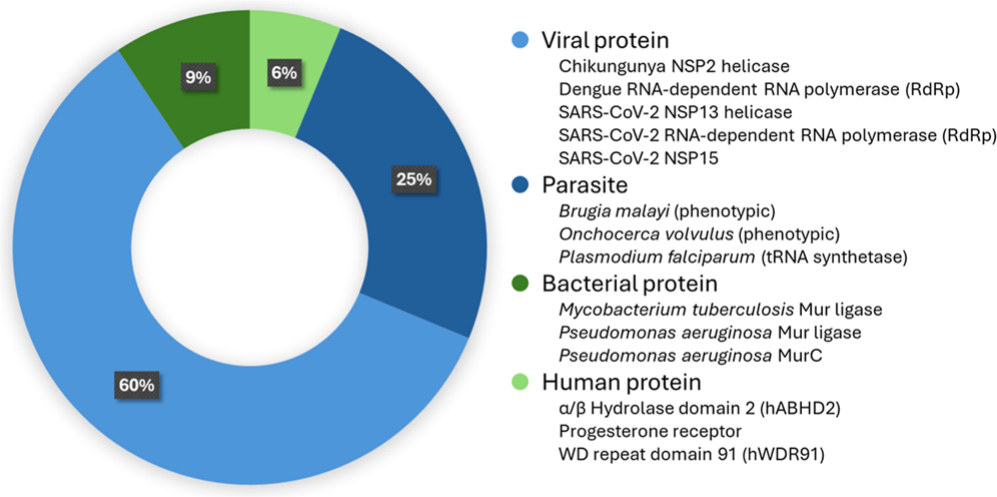
Targets for which the compounds on plate MHT-0001 were originally intended. The shared plate contained compounds from various projects focusing on viral (60% of compounds), parasitic (25%), bacterial (9%), and human (6%) targets; most projects were target-based rather than phenotypic.

The functional group diversity of the plate can be easily monitored (Figure 4 and Supporting Information) to ensure broad coverage. The diversity of the compounds was also characterized using both 2D descriptor and 3D descriptors (Figure 4) to assess drug-likeness and predict oral bioavailability. Both types of descriptors are typically more useful in the later stages of compound development but can act as rough guides in earlier stages. Compound 2D descriptors are physicochemical properties, such as the Lipinski guidelines^17,18^ with which the compounds in the plate align well. The 3D descriptors, which have become more widely used in the past 15 years, address the predicted topological polar surface area (TPSA) and the fraction of sp^3^ hybridized carbons.^19,20^ As the compounds selected on the plate are still in the early stage of development, and as there can be healthy disagreement as to the utility of individual compound metrics,^21^ it is helpful if plates aim for a diversity of values.

**Figure 4:**
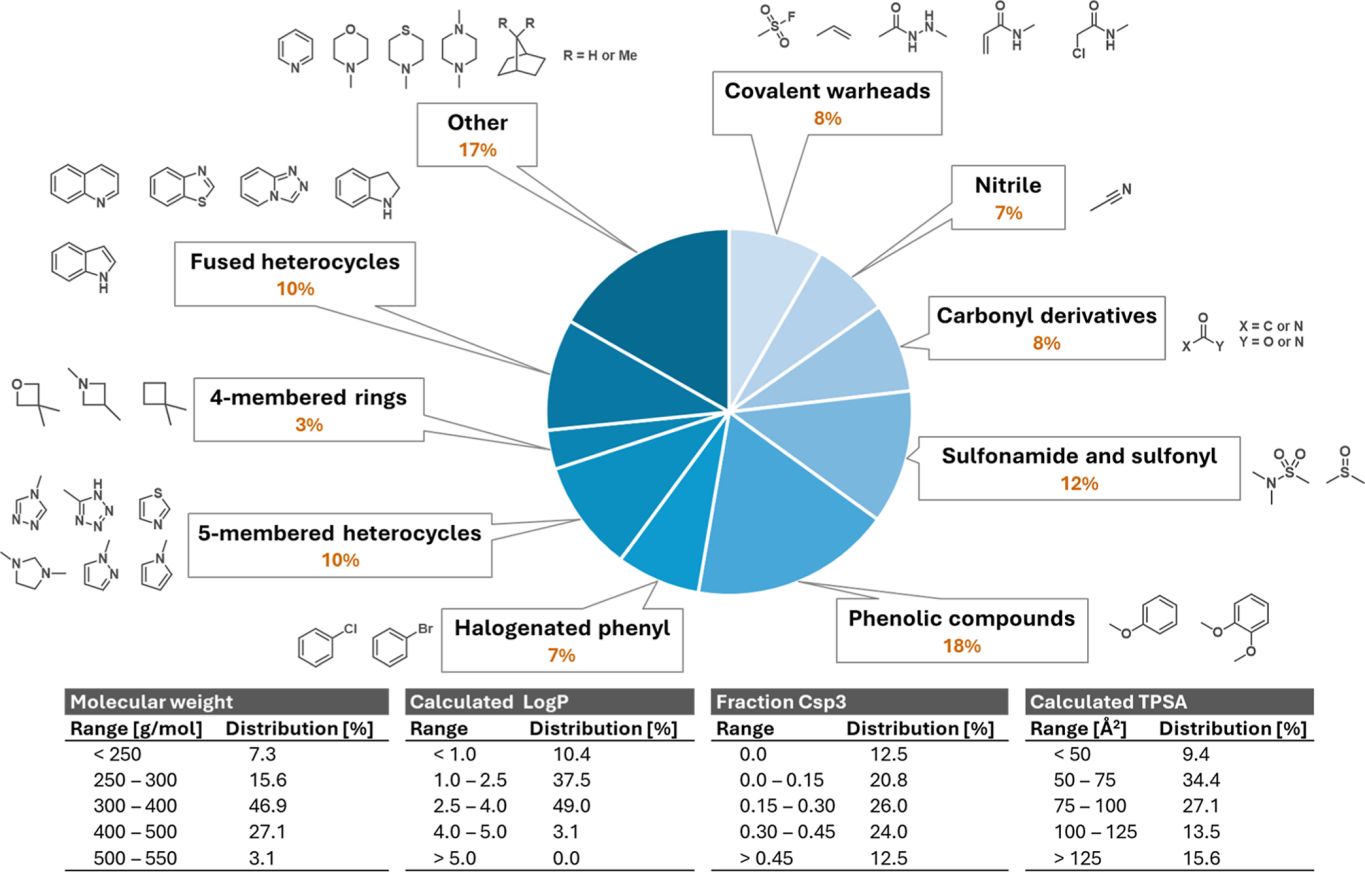
Compounds of plate MHT-0001 were selected to be structurally diverse. A range of functional groups is represented, and most compounds align with desirable values of descriptors commonly used in medicinal chemistry programmes.

The compounds were checked for potential risk motifs that can cause pan-assay interference using the SwissADME online tool.^22^ Among the compounds selected for the plate MHT-0001, 10 contain known motifs usually intended to form covalent bonds, and these were unsurprisingly highlighted in this analysis, along with five compounds that have the potential to act as covalent inhibitors under suitable conditions. Only two compounds were of concern, one displaying a hydantoin and the other a Mannich phenol. The compound bearing the latter motif has been shown not to act as a pan-assay inhibitor in the original project,^23^ while the former compound was derived from a commercial HTS library and showed no inhibition when tested in the original project-relevant assays (dengue RdRp biochemical and whole cell antiviral assays).^24^ Inclusion of covalent or potentially promiscuous motifs would be at the discretion of the team assembling the library or plate.

### 1.5 Where to Send Idler Compounds

There are likely to be many laboratories interested in evaluating your idler compounds, though these can change over time and the nature of the collaboration arrangement will vary. Academic laboratories may be funded to identify new hits targeting biologically important proteins, but frequently this capability is limited by the term of a grant; nongovernmental organisations (NGOs) can often act as honest brokers between laboratories supplying chemical matter and suitable biologists. One of the best-known laboratories is the US National Cancer Institute (NCI) that uses 60 different human tumor cell lines to screen compounds, but recently this service has been made available only from within the United States.^25^ The Community for Open Antimicrobial Drug Discovery (CO-ADD), though no longer funded, continues to screen community submissions for antibacterial and antifungal activity.^26^ The more recent EU-OPENSCREEN project will accept compound submission and will evaluate those molecules in a range of physicochemical and biological assays free of charge and in a way that provides the submitter control of the use of any arising intellectual property.^27^ Exscalate4CoV focuses on identifying new therapies against the SARS-CoV-2 virus.^28^ At UKHSA, the Open Innovation Platform for early stage drug discovery against infectious diseases will consider applications to screen compounds against defined priority pathogens,^29^ as will UKHSA’s program for Discovery Partnerships.

However, there is a notable lack of clear and consistent long-term options for compound evaluation, despite the possible attendant benefits for important areas such as pandemic preparedness. By highlighting the potential use of idler compounds and outlining a simple protocol for assembling a suitable plate, we hope to demonstrate the healthy state of the demand side of this equation to stimulate solutions for the supply of compound evaluation.

## 2 Results and Discussion

Compounds in plate MHT-0001 were evaluated at UKHSA against a selection of bacterial strains (*Staphylococcus aureus*, *Acinetobacter baumannii*, *Escherichia coli*, *Mycobacterium abscessus*, and *Mycobacterium avium*) and one fungal strain (*Candida albicans*), as outlined in the methods section (though 9% of the tested compounds were intended as antibacterials, that design was primarily target-based, rather than phenotypic; there is value in evaluation of such compounds more broadly, but there is also no harm in including some compounds as potential controls, to guard against differences in assays). To address potentially false positive results due to aggregation and solubility issues, the compounds on the plate were experimentally evaluated for solubility. Predictive algorithms for solubility can be challenging to use.^30^ However, there are many commercial vendors of solubility measurements. There are no simple generic ways to measure solubility on milligram quantities of material in-house in the academic laboratory, though the relevant equipment is becoming more accessible (e.g., the NEPHELOstar Plus from BMG LABTECH). While aggregation data is also useful and important when assessing biological activity of compounds,^31^ this is a costly and not easily accessible test for most academic medicinal chemistry laboratories unless accessed through collaborations or use of a contract research organization (CRO).

Comparing the inhibitory activity of the compounds on *S. aureus*, *A. baumannii*, and *E. coli* showed 12 compounds with activity on Gram-positive *S. aureus* (Supporting Information), while only one compound showed activity on a Gram-negative strain (compound WEL2.92a in well G8, Figure 5A). Permeabilising the outer-membrane of the Gram-negative bacteria *A. baumannii* and *E. coli* with polymyxin b nonapeptide (PMBN)^32^ increased the uptake of compounds and revealed several compounds with narrow–spectrum activity on either *A. baumannii* (well C6, Figure 5A) or *E. coli* (well E7, G6, H5, and H12, Figure 5A). Compound WYH-9 in well H12 was initially developed to target Gram-negative bacterial Mur ligases as part of the Open Source Antibiotics project;^33^ Mur ligases are vital for building and maintaining the peptidoglycan structure of both Gram-positive and -negative bacteria. Several of the tested MHT-0001 compounds present broad-spectrum activity on both Gram-negative and Gram-positive bacteria (Figure 5A and Supporting Information). Notable among these is compound **WEL2.76** (well G9 in Figure 5A,B) that shows strong activity versus *S. aureus*, *A. baumannii* (+PMBN), and *E. coli* (+PMBN), with no noticeable effect on *C. albicans* or the two mycobacterium strains (Figure 6A and Supporting Information). This compound was initially developed for a *P. falciparum* project and shows promising antiplasmodial activity.^34^ In this cross-screen we have shown that this compound also presents additional antibacterial activity. Future work would involve improving the solubility of the compound as it is poor (Figure 6B and Supporting Information).

**Figure 5:**
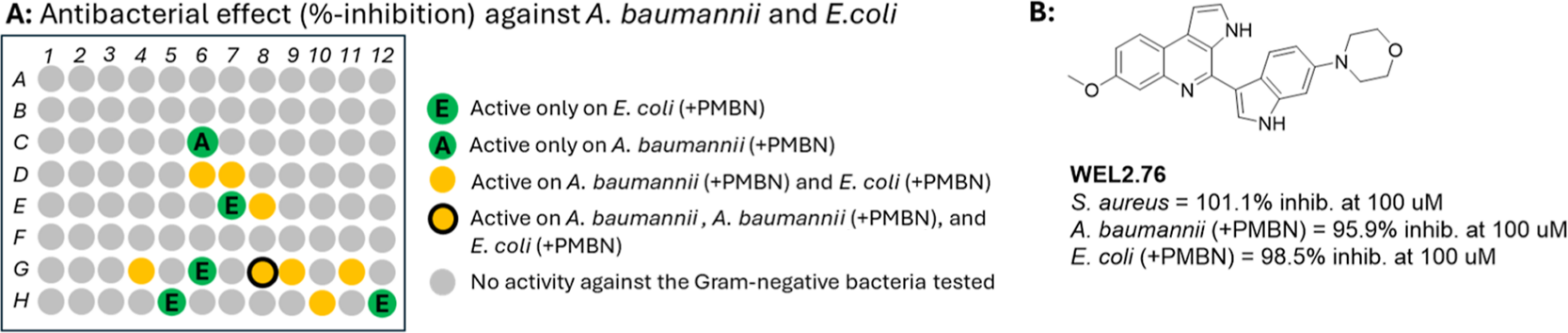
(A) Comparison of antimicrobial activity against *A. baumannii* (∓PMBN) and *E. coli* (∓PMBN). Compounds are reported as active when inhibition is >80% at 100 μM compound concentration. (B) Chemical structure and antimicrobial activity of **WEL2.76**.

**Figure 6:**
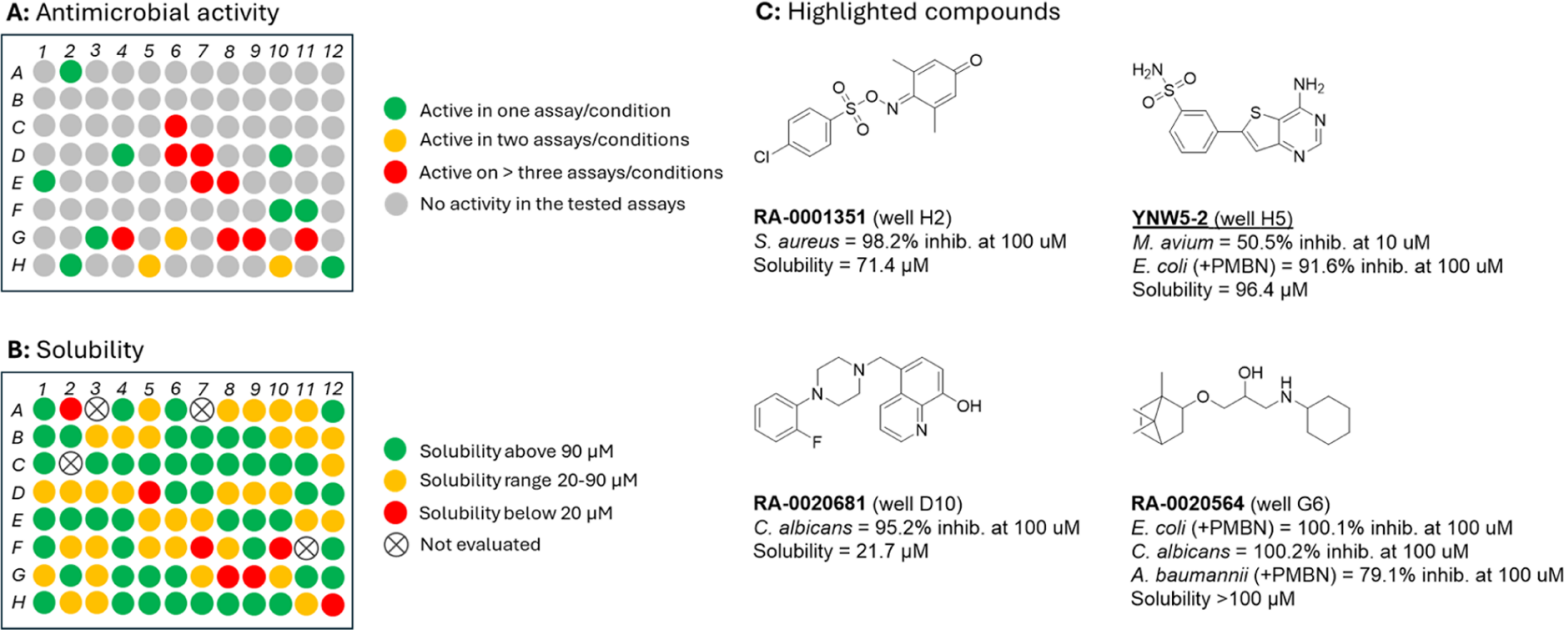
(A) Antimicrobial activity against *S. aureus*, *A. baumannii* (∓PMBN), *E. coli* (∓PMBN), *C. albicans*, *M. avium*, and *M. abscessus*. Compounds are reported as active when inhibition is >80% at 100 μM compound concentration (*S. aureus*, *A. baumannii* (∓PMBN), *E. coli* (∓PMBN), *C. albicans*) and >50% inhibition at 10 μM compound concentration (*M. avium*, and *M. abscessus*). (B) Solubility of the compounds. (C) Chemical structures of the highlighted compounds.

Another notable compound is **RA-0001351** (well H2 in Figure 6A,C), that presents with a reactive iminoquinone core yet shows very good selectivity for *S. aureus* over all other tested microbes (Figure 6A), as well as good solubility (Figure 6B and Supporting Information). This compound was developed as part of a SARS-CoV-2 nonstructural protein 12 (Nsp12) project, and, while potent on Nsp12, it was also highly active in a glutathione (GSH) assay. Extensive work identified an unexpected mechanism for the reaction between **RA-0001351** and GSH.^35^ Our initial concern that this compound would be promiscuous was lessened by its selective activity in this cross-screen.

The compounds on the plate were also evaluated for antifungal activity on *C. albicans* (Supporting Information), with ten compounds inhibiting growth. Three showed selective activity toward *C. albicans* (wells D10, F11, and G3 Figure 6A) over the bacterial strains tested. Compound **RA-0020681** (well D10 Figure 6A) was originally designed for the chikungunya virus nsp2 helicase^36^ and was one of 16 compounds purchased for further follow-on analysis after a high-throughput screen (HTS) of an Asinex commercial library but showed no unwinding or ATPase inhibitory effect. This idler compound now shows promise as a selective antifungal agent that warrants further investigation; solubility was poor, and further work would be needed to improve this (Figure 6B).

Finally, the compounds were screened in *M. abscessus* and *M. avium* assays. The compounds were run at a lower concentration (10 μM cf. 100 μM for the other assays; these are used as standard by the collaborating team), and the cutoff for a compound to be denoted as active was set at 50% inhibition (cf. 80% for the other assays). Only one compound showed activity according to this criterion: **YNW5-2** (well H5 in Figure 6A,C). This compound was originally synthesized as part of the Open Source Malaria project, where it displayed potent and selective inhibition against *P. falciparum* via inhibition of at least one tRNA synthetase enzyme;^16^ it has previously been screened against a selection of bacterial and fungal targets.^37^ Of the eight other compounds from this same series included in the plate, only one showed moderate activity: **YNW88**, an analogue of **YNW5-2** with 45% inhibition at 10 μM, also against *M. avium*. The other analogues showed negligible inhibition at 10 μM. The solubility of all the YNW analogues was very good (>90 μM, Figure 6B and Supporting Information).

This cross-screen also highlighted a few compounds with activity on several strains tested. One such compound is **RA-0020564** (well G6 in Figure 6A,C) with activity on both Gram-negative bacteria (*A. baumannii* + PMBN, *E. coli* + PMBN) and *C. albicans*. This compound was part of project focused on dengue RNA-dependent RNA polymerase (DENV RdRp); the molecule was one of 114 compounds purchased as a follow up to a high-throughput screening campaign of a LifeChemicals 50k diversity library.^24^ This campaign only generated one compound with suitable antiviral activity, but without a confirmed target (RA-0020572; 2.7 μM on DENV serotype 2), and thus 16 of these idler compounds were included in this cross-screen. However, the only compound of note was the promiscuous **RA-0020564**.

It is important to evaluate toxicity to provide context for any phenotypic activity. The compounds were thus tested in a hemolysis assay, which is one measurement of toxicity, and no compounds showed any lysis of red blood cells (RBCs) in this assay (Supporting Information). For a small selection of compounds, separate cytotoxicity testing has also been carried out as part of the originating project and known problematic compounds should normally be excluded from the library. Newly acquired data can obviously influence interpretation of screening results: in the case of **RA-0020564**, this compound was reported as cytotoxic in Huh7 cells (CC_50_ = 14.5 μM)^24^ after plate MHT-0001 was prepared.

The above data are shared directly, as received, on the ELN, along with protocols and clear attribution to the relevant researchers; subsequent plates can then be formulated and added. The data are also curated into an online sheet, making data analysis easier; the data may be downloaded in commonly used formats. The data have been shared to other widely available online platforms such as Zenodo,^38^ PubChem,^39^ and a university e-repository,^40,41^ to ensure longevity and to avoid data becoming unavailable due to commercial suppliers going out of business or through “link rot”. If sufficient data are generated, uploading to larger repositories such as ChEMBL^42,43^ or PubChem Bioassays^44^ could be considered, though this requires a little more dedicated effort. Sharing data to such repositories will allow for collaborative data to be generated on single hits, e.g., to check for promiscuity, or unexpected activity. Employing a simple license, such as CC-BY,^14^ makes it clear to all readers what their freedoms are in using the data in their own work. This roadmap therefore promotes a grassroots effort in compound evaluation and data sharing, where researchers contribute their data to shared, online repositories in a way that better leverages the value of idler compounds, toward new collaborations and publications. We thank a referee for suggesting that perhaps those online data repositories may be willing to highlight data sets that have been contributed this way.

## 3 Conclusions

Obtaining maximum scientific value from existing resources and embracing serendipity in research are worthwhile. Doing such things openly will benefit everyone. It is therefore likely to be productive to get your idler compounds moving, but the question is “how?”. We hope that this short how-to guide provides some simple solutions to enabling open discovery with the least resource cost.

We wish it were easier. While there are many available electronic laboratory notebooks, few make it easy to share data publicly. Given the usefulness of biologically evaluating compounds and the overhead in setting up such resources, there are few long-term investments made into such facilities. Despite the potential benefits of high-quality, machine-readable data for machine learning-enabled insights into predictive drug discovery, there is currently little momentum toward home-grown accumulation of relevant, open, unencumbered data.

For us, it was worth it and remains so. We have serendipitously discovered new activity that is now being followed up toward new papers and grant proposals. Group members contributed to a shared good that has generated additional data, and which encourages shared care in the creation of a valuable resource. Our funders will, we hope, be pleased we have extracted additional value from their support. We are looking forward to assembling subsequent boxes and sharing the data online, and would invite you to join in.

## 4 Experimental Section

### 4.1 Assay to Test Activity against *S. aureus*, *A. baumannii*, and *E. coli*

Activity of compounds was assessed against pathogenic bacteria using a method adapted from the EUCAST guidelines for microbroth dilution minimum inhibitory concentration (MIC) assays.^45^ *S. aureus* (ATCC 9144), *A. baumannii* (ATCC 17978) and *E. coli* (NCTC 12923) were maintained on Tryptic Soy Agar (TSA) plates for up to 2 weeks. Overnight cultures were grown in cation-adjusted Mueller Hinton Broth (CAMHB) by picking colonies from TSA plates and back-diluting to a concentration of ∼5 × 10^5^ CFU/mL in CAMHB. 198 μL of these cultures were added to 2 μL of 10 mM compounds, providing a final concentration of 100 μM compounds, and incubated at 37 °C for 20 h. The OD_600_ was read using a CLARIOstar Plus plate reader (BMG LABTECH). A “hit” was defined as a compound which inhibited 80% or more of growth compared to an untreated control. DMSO-only controls were run alongside and did not impact growth.

As penetration into Gram-negative bacteria is often the rate-limiting step in discovery of novel antimicrobial compounds against these pathogens, an additional assay was performed for the Gram-negative bacteria, where the above assay was performed in the presence of polymyxin b nonapeptide (PMBN). PMBN was added at a fixed concentration of 30 μg/mL to permeabilise the outer membrane.^32^

### 4.2 Assay to Test Activity against *C. albicans*

Activity against *C. albicans* (NCPF 3281) was determined using an adapted protocol based on EUCAST guidelines for microbroth dilution MIC assays for yeast.^46^ *C. albicans* was maintained on Sabouraud agar (SAB) plates for up to 2 weeks. Overnight cultures were grown in RPMI medium supplemented with 2% glucose, adjusted to pH 7, and back diluted to a concentration of ∼5 × 10^5^ CFU/mL. 198 μL of this culture was added to 2 μL of 10 mM compounds, providing a final concentration of 100 μM compounds, and incubated at 37 °C for 24 h. The OD_530_ was read using a CLARIOstar Plus plate reader (BMG LABTECH). A “hit” was defined as a compound which inhibited 80% or more of growth compared to an untreated control. DMSO-only controls were run alongside and did not impact growth.

### 4.3 Assay to Test Activity against *Mycobacterium abscessus* and *Mycobacterium avium*

*Mycobacterium abscessus* (NCTC 14045) and *Mycobacterium avium* (ATCC15796) strains were transformed with a plasmid expressing a bacterial luciferase, pMV306hsp + LuxG13. The reporter strain contained the luxABCDE operon and a G13 promoter. Middlebrook 7H9 broth (10% OADC, 0.5% glycerol, 0.2% Tween 80) was prepared and stored at 4 °C until required. 1.0 mL of defrosted culture was used to inoculate 25 mL of Middlebrook 7H9 broth (supplemented with OACD) in an Erlenmeyer flask and incubated at 37 °C while shaking at 200 rpm for either 24 h (*M. abscessus*) or 7 days (*M. avium*). Following the incubation period, the cultures were diluted with phosphate-buffered saline (PBS, pH 7.4) until the OD_540_ was 0.05 for both *M. avium* and *M. abscessus*. Plates containing 1 μL of the prediluted compounds at 1 mM (as outlined in Figures S1 and S2) were thawed to room temperature then diluted with culture, providing a final concentration of 10 μM for each compound (unlike 100 μM used in other assays; compounds without activity at 10 μM in this assay have rarely been found to be worthy of follow-up). DMSO at a concentration of 0.1% (v/v in medium) was used as a negative control, while 16 μM of bedaquiline^47,48^ was included as a positive control. Plates were incubated at 37 °C and while shaking at 200 rpm for their respective incubation period (24 h or 7 days) and luminescence was measured at 540 nm using a GloMax Discover plate reader (Promega, Figure S3). A “hit” was defined as a compound which inhibited 50% or more of growth compared to an untreated control.

### 4.4 Hemolysis

An indication of the cytotoxicity of the compounds was determined by incubating them at 100 μM with human red blood cells (RBCs) and measuring the level of RBC lysis, as performed previously.^49^ RBCs were collected from healthy volunteers and used within 24 h of collection. RBCs were isolated from whole blood by centrifugation, washed in PBS three times and resuspended in PBS to 10% v/v. RBCs were added to a V-bottom polypropylene plate containing compounds and appropriate controls, and incubated at 37 °C for 1 h. RBC were then pelleted by centrifugation and the supernatant was carefully transferred to a fresh 96-well plate. The OD_550_ was measured using a CLARIOstar Plus plate reader (BMG LABTECH). The % hemolysis was calculated as follows: % hemolysis = (*A*_p_ – *A*_B_)/(*A*_c_ – *A*_B_) × 100, where *A*_p_ is the absorbance for each compound, *A*_B_ is the absorbance of the 0.1% Triton-X-100 positive lysis control and *A*_c_ is the absorbance for the PBS negative lysis control. DMSO controls were run alongside and did not demonstrate any lysis of RBCs.

### 4.5 Solubility

The 10 mM DMSO stock solutions of plate MHT-0001 were diluted to a series ranging from 100 μM to 0.1 μM in a 384 well transparent plate (Greiner 781801) with a final concentration of 1% DMSO in DPBS (Sigma D8537, pH = 7.1). The compounds were incubated at 37 °C and the forward light-scatter was measured using a NEPHELOstar Plus (BMG LABTECH) after 2 h. During analysis the results were adjusted to a segmented regression to obtain the maximum concentration in which compounds are soluble.

## Supplementary Material







## Notes

A guide of library generation, links to additional online material, chemistry synthesis information and supporting biological protocols (PDF)
Compound ID names and SMILES (CSV)
The assay results including compound details (SMILES, molecular weight), the structural motif analysis of the selected compounds, and the 2D and 3D descriptors of the compounds (XLSX)

## Abbreviations

CAMHB: cation-adjusted Mueller Hinton Broth
CC-BY: Creative Commons Attribution License
CFU: colony-forming unit
CO-ADD: community for open antimicrobial drug discovery
CRO: Contract Research Organization
DPBS: Dulbecco’s phosphate-buffered saline
ELF: European Lead Factory
ELN: electronic laboratory notebook
EUCAST: European Committee on Antimicrobial Susceptibility Testing
FAIR: findable, accessible, interoperable and reusable
GSH: glutathione
NGO: nongovernmental organization
Nsp: nonstructural protein
OADC: oleic acid, albumin, dextrose, and catalase
PMBN: polymyxin b nonapeptide
SARS-CoV-2: severe acute respiratory syndrome coronavirus 2
TPSA: topological polar surface area
TSA: tryptic soy agar
UKHSA: UK Health Security Agency
