# Supplementary material for: Idler Compounds: A Simple Protocol for Openly Sharing Fridge Contents for Cross-Screening
Source: J Med Chem. 2026 Jan 28;69(3):2453–61. doi: 10.1021/acs.jmedchem.5c02354 (PMC12928571; doi:10.1021/acs.jmedchem.5c02354)
Supplement: Supplementary file 1 [file jm5c02354_si_001.pdf]

Supplementary Information for

Idler Compounds: A Simple Protocol for Openly Sharing Fridge Contents for Cross-Screening

Rebecka Isaksson[1†], Eve M. Carter[1†], Charlotte K. Hind[2], J. Mark Sutton[2], Hazel Rudgyard[3], Adam H. Roberts[3], Christopher W. Moon[3], Yinuo Wang[1], Todd Group Researchers[1], Sandra Codony[4,5], Antón L. Martínez[6], Joanna Bacon[3], Matthew H. Todd[1]*

[1] UCL School of Pharmacy and Structural Genomics Consortium, 29-39 Brunswick Square, London WC1N 1AX, United Kingdom.

[2] Antimicrobial Discovery, Development and Diagnostics, Countermeasures, Development, Evaluation and Preparedness, UK Health Security Agency, Salisbury SP4 0JG, United Kingdom.

[3] Discovery Group, Countermeasures, Development, Evaluation and Preparedness, UK Health Security Agency, Salisbury, SP4 0JG

[4] Institute of Biomedicine of the University of Barcelona (IBUB), Universitat de Barcelona, 08028 Barcelona, Spain.

[5] Laboratori de Química Farmacèutica, Facultat de Farmàcia i Ciències de l'Alimentació, Universitat de Barcelona, Av. Joan XXIII, 27-31, 08028 Barcelona, Spain.

[6] Innopharma Drug Screening Platform/Biofarma Research Group, CIMUS Research Center, Pharmacology Department, School of Pharmacy, University of Santiago de Compostela (USC) and Instituto de Investigación Sanitaria de Santiago de Compostela (IDIS), Santiago de Compostela 15782, Spain.

† Authors contributed equally

* To whom correspondence should be addressed, matthew.todd@ucl.ac.uk

The Todd Group Researchers who contributed to this work are (in alphabetical order): Mohsen Alamoudi, Hadia Almahli, Guilherme Fernandes, Kangping Liu, Alvaro Magalhaes, Evans Mainsah, Xin Qui, Rahman Shah Zaib Saleem, Wellington da Silva, and Yuhang Wang.

‡ To whom correspondence should be addressed, matthew.todd@ucl.ac.uk

Contents

Access to online idler library and additional data S2
Step-by-step guide to library generation, selection, and analysis S3
Shipping and tracking compounds S5
General chemistry S6
Supporting biological protocols S6
Preparation of Assay Plates for *Mycobacterium abscessus* and *Mycobacterium avium* S6
Diagram of the growth process and testing against *M. absessus* and *M. avium* S7
References S8

Access to online idler library and additional data

The full Todd Group Idler Library can be viewed here: https://tinyurl.com/yynht8mu.

Accompanying data is found in the Todd Group Idler Library LabArchive electronic lab notebook: https://tinyurl.com/3jf88u32.

To learn more about the project that a compound originated from (including synthetic protocols in applicable cases), please visit the respective GitHub repository. In the Todd Library Google spreadsheet (https://tinyurl.com/yynht8mu) find the GitHub code listed in the document. Search for GitHub + Code – for example: "GitHub OSM Series 3" will take you to the Open Source Malaria Series 3 GitHub repository.

Should you not find the information you are looking for, please use the Issues tab on the repository or reach out to the corresponding author.

All the biological data and the chemical 2D and 3D properties have been shared with this publication as a supplementary document (separate excel sheet).

We also share all the data generated in the assays mentioned in this publication via the following sources:

- PubChem: https://pubchem.ncbi.nlm.nih.gov/bioassay/2061108
- Zenodo: https://doi.org/10.5281/zenodo.15409475
- UCL Library Snapshot: https://doi.org/10.5522/04/29881166.v1

Step-by-step guide to library generation, selection, and analysis

Generating an online library that is sharable and searchable

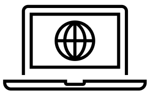

To ensure compounds are added to the library in the same format, we have established a protocol that is available to view but summarised here:

- The online spreadsheet that contains the overview of the library holds several columns that each researcher is asked to fill in; these include physical location of vial containing compound, submitter details including project the compound was designed for, strings for compound structure, as well as physiochemical properties.
- Compounds are assigned individual codes that are unique to the project it originates from, but since compounds are often also tied to a lab notebook experiment and related code this is also mentioned in the spreadsheet.
- If a compound is resynthesised or repurchased, the new batch is added as new entry in the spreadsheet, often denoted with -02 to mark the second batch, with –01 being the original.

Compound handling and quality control

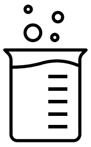

- Compounds in the library are generally designed to follow the Lipinski Ro5 guidelines[1,2], but as noted in Figure 4 in the main paper some more developed compounds in the library exceed these guidelines.
- Synthesised compounds are analysed with standard protocols (LCMS, NMR, melting point, etc.) as applicable to determine and confirm structure and purity.
- Synthesised compounds with purity above 90% on LCMS and ^{1}H NMR are added to the library.
- Purchased compounds are added directly to the library if a quality control has already been performed by the supplier or analysed with only LCMS if not. If LCMS is needed, we find it saves time to arrange quality control samples as the stock solutions are prepared.
- The Lipinski properties, such as molecular weight, predicted logP, and H-bond donors and acceptors, can be calculated using open software such as DataWarrior.
- Compound properties can be evaluated using online tools such as SwissADME, but tend to be slower for large sets of compounds[3]. The benefit for this online tool is that is also provides information about TPSA and fraction of sp3 carbons – two 3D properties used to predict compound performance. For our library, we used DataWarrior[4] (v05.05.00) and the built-in function *Calculate Properties* as outlined below to calculate these properties for the compounds:

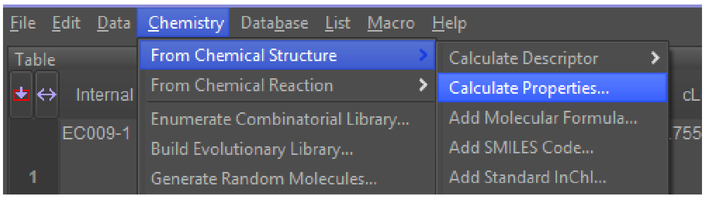

Sample preparation

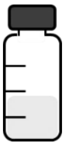

Stock solution preparation:

- Compounds are weighed out in glass vials and dissolved in biological grade DMSO (Sigma Aldrich; cat. D8418) to a final concentration of 10 mM*.
- The vials are stored in suitably marked boxes at –20 °C until needed[5].

Plate preparation:

- For selected compound, the vials are collected from their respective boxes and thawed at 30 °C.
- An aliquot of each compound (e.g. 50 µL, depending what is required) is added to a respective well on the 96-well plate (ThermoFisher Scientific; cat. 249944), and the plate is sealed with a DMSO-resistant foil (Sigma Aldrich; cat. BR701367).
- The plate is frozen in preparation of shipment (process outlined in appendix 2).

* Three stock solutions in the library holds a lower concentration.

Compound selection

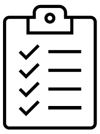

For plate MHT-0001, researchers were asked to nominate compounds (56 in total), and the remaining wells on the plate was filled from the commercially available compounds that had been added to the library:

- The commercial compounds were divided into 40 clusters based on chemical similarity, using a DataWarrior worksheet and the built-in clustering tool *Cluster Compounds/Reactions*.
- The limit was set to when 40 clusters reached; this tool is based on a chemical similarity search where the tool can be limited to the % similarity in each cluster or the number of clusters, in which case the similarity will vary between clusters.
- After generating the 40 clusters, a representative from each cluster (listed by DataWarrior) was selected for the 96-well plate.

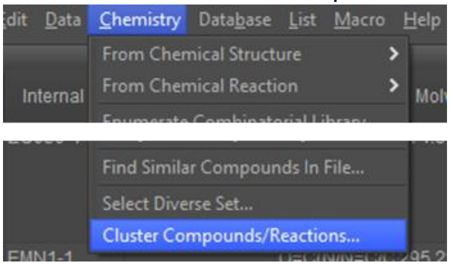

Once all compounds had been selected, the full 96 compounds were checked for similarity to ensure good diversity on the plate.

- Using Datawarrior and the clustering tool descried above, a similarity search was selecting that a new cluster is generated if the similarity fell below 80%.
- There were 12 compounds with more than 80% similarity, but after manual inspection, it was decided to only exchange one compound for another with greater structural variation.
- Before sharing the plate, an accompanying spreadsheet was prepared listing the compounds and their properties, as well as location on the plate. A template can be downloaded here: https://tinyurl.com/3jf88u32.

Plate analysis

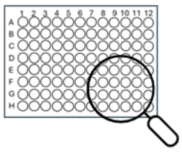

The diversity of the plate was analysed both through counting drug-like properties (like the 2D properties of Lipinsky Ro5[1,2], as well as 3D properties[6,7] such as fraction Csp3 or calculated PSA) as well as through a functional group analysis. This was performed using DataWarrior and Excel as outlined below:

- Functional group prevalence in each molecule is counted by searching for individual motifs that are distinguishing for the compounds; the function *Add Substructure Count* is useful:

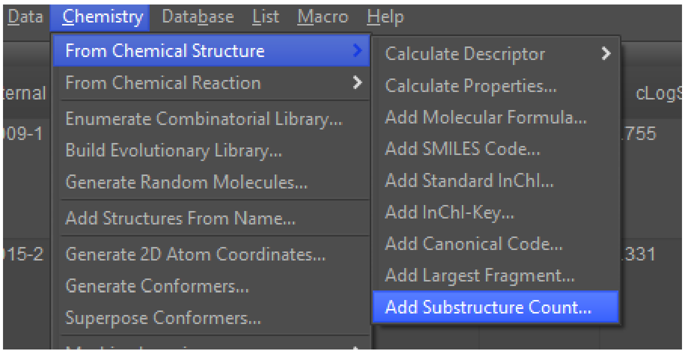

- After minor curation (ensuring groups are not counted in error, e.g. a carbamate would be counted as an amide erroneously), the resulting spreadsheet is analysed using Excel to determine the count of each functional group in the subset selected for the plate.
- The Lipinski Ro5 parameters is calculated using DataWarrior as outlined above and transferred to Excel where the percentage per range is determined (using the COUNTIFS function).
- There are several free online tools available to analyse the compounds for the druglikeness; SwissADME[3] is one such tool that also lists potentially problematic structural motifs that tend to provide false positives (PAINs). This tool was used to analyse the compounds selected for the plate and the potential problematic structures.

Shipping and tracking compounds

Sending plates of compound is essentially very straightforward, and it is beneficial to keep an internal record of which recipient have received what material. Our process is outlined in the ELN (https://tinyurl.com/3jf88u32) and summarized here:

- Once the plate is ready to send, it is shipped on dry ice (with suitable packaging).
- We use an online communal tracking index, where the plate number is connected to a tracking number and destination.
- With every shipment, the spreadsheet for the plate layout (mentioned above in compound selection) is attached along with other necessary documentation.

General chemistry

The Todd Library consists of a mix of synthesised and purchased compounds from commercial libraries. **Synthesised compounds**: Synthetic procedures and compound characterisation is reported in the original ELN, accessible through the relevant Github repository. Reagent and solvents were purchased from standard suppliers and used without further purification. Products were purified using automated column chromatography (Biotage Selekt and Isolera flash purification systems with Biotage Sfär Duo normal phase or C18 columns), or reverse-phase high-performance liquid chromatography (Agilent Infinity 1290 II system consisting of an isocratic pump (G7110B) and a diode array detector WR (G7165A) coupled to an InfinityLab LC/ MSD (G6125B) using ESI and a fraction collector (G7159B). An Agilent Eclipse XDB-C18 column (7 µm, 21.2 mm × 250 mm) was used with a mobile phase of 0.05% formic acid in H_2O and 0.05% formic acid in MeCN). Products were analysed with LCMS (Agilent Infinity 1260 II system consisting of a quaternary pump (G7111A) and a diode array detector WR (G7115A) coupled to an InfinityLab LC/ MSD (G6125B) using ESI. An Agilent Poroshell 120 EC-C18 column (2.7 µm, 4.6 mm × 50 mm) was used with a mobile phase of 0.05% formic acid in H_2O and 0.05% formic acid in MeCN) and ^{1}H and ^{13}C NMR (Bruker Avance III 400) to confirm >90% purity. **Purchased compounds**: Compounds were obtained from commercial libraries provided by Enamine, Chemspace, LifeChemicals, ChemDiv, and Molport. The compounds were delivered as solids to a purity of >90% and used without further analysis. Compounds of interest were then analysed by LCMS to confirm purity.

Purity of compounds derived from re-supply will be of the same purity as the original sample unless resynthesised, and unless the compound is commercially available.

Supporting biological protocols

Preparation of Assay Plates for *Mycobacterium abscessus* and *Mycobacterium avium*

The master 96-well plate MHT-0001 contained 96 compounds at a concentration of 10 mM in DMSO and volume of 50 µl. Using PBS, a 1:10 dilution was completed to produce two plates of 1 mM, one for each microbial species (Figure S1). From these plates, 1 µl was transferred from each well into one of two separate plates to allow space for positive and negative controls (Figure S1 and S2). Plates were stored at −80 °C until required. During the assay, this was diluted to the desired final concentration of 10 µM (Figure S1).

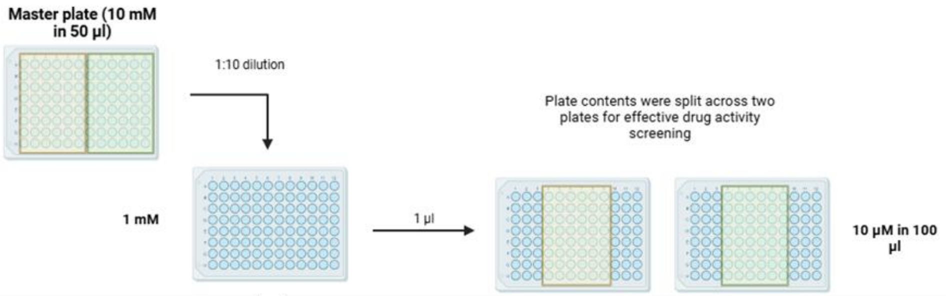

Figure S1: Schematic illustrating plate preparation for assays at 10 µM concentrations. Dilutions using PBS were initially carried out to obtain plates of concentrations 1 mM as shown on the left. These plates were then split and further diluted.

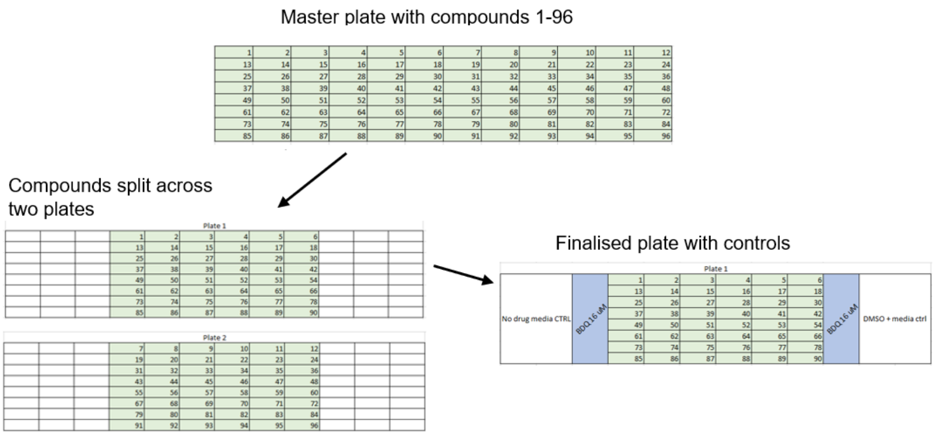

Figure S2: Positional layout of all compounds in 96-well plates. Compounds were split down the centre of the master plate, with compounds in columns 1-6 placed in plate 1 whilst columns 7-12 in plate 2, as shown on the left. Final plates included a Bedaquiline positive control either side, followed by negative controls containing no addition of compound.

Diagram of the growth process and testing against *M. abscessus* and *M. avium*

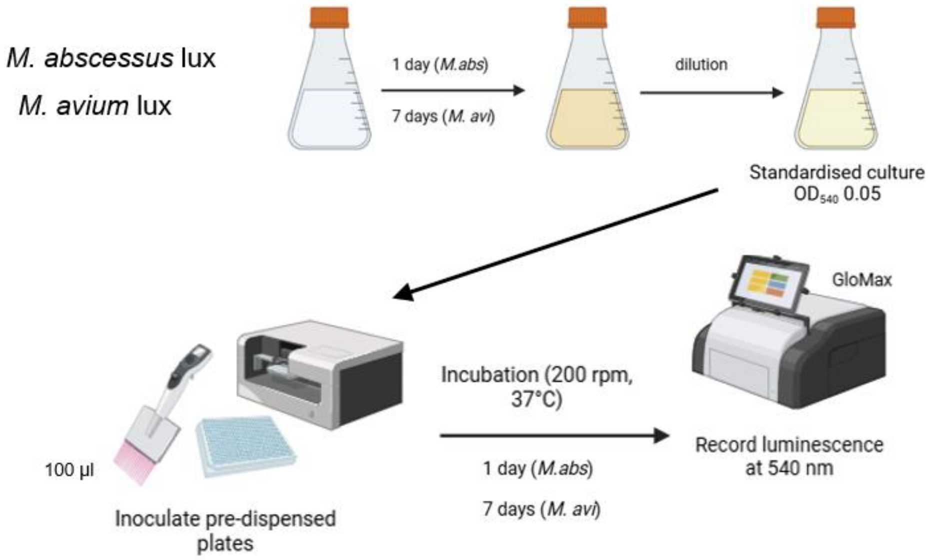

Figure S3: Culture growth and drug assay for *M. absessus* and *M. avium*. The diagram outlines the main steps, starting with inoculation of culture in Middlebrook 7H9 broth, followed by culture dilution, plate-inoculation, and recording of luminescence at 540 nm.

References

1. Lipinski, C. A.; Lombardo, F.; Dominy, B. W.; Feeney, P. J. Experimental and Computational Approaches to Estimate Solubility and Permeability in Drug Discovery and Development Settings. *Adv. Drug Delivery Rev.* **46** (1−3), 3−26 (2001).

2. Lipinski, C. A. Lead- and drug-like compounds: the rule-of-five revolution. *Drug Discov. Today Technol.* **1**, 337–341 (2004).

3. Daina, A., Michielin, O. & Zoete, V. SwissADME: a free web tool to evaluate pharmacokinetics, drug-likeness and medicinal chemistry friendliness of small molecules. *Sci. Rep.* **7**, 42717 (2017).

4. Sander, T., Freyss, J., von Korff, M. & Rufener, C. DataWarrior: An Open-Source Program For Chemistry Aware Data Visualization And Analysis. *J. Chem. Inf. Model.* **55**, 460–473 (2015).

5. Ilouga, P. E., Winkler, D., Kirchhoff, C., Schierholz, B. & Wölcke, J. Investigation of 3 Industry-Wide Applied Storage Conditions for Compound Libraries. *SLAS Discov.* **12**, 21–32 (2007).

6. Kombo, D. C. *et al.* 3D Molecular Descriptors Important for Clinical Success. *J. Chem. Inf. Model.* **53**, 327–342 (2013).

7. Lovering, F., Bikker, J. & Humblet, C. Escape from Flatland: Increasing Saturation as an Approach to Improving Clinical Success. *J. Med. Chem.* **52**, 6752–6756 (2009).
